# Lipophilicity of Bacteriochlorin-Based Photosensitizers as a Determinant for PDT Optimization through the Modulation of the Inflammatory Mediators

**DOI:** 10.3390/jcm9010008

**Published:** 2019-12-19

**Authors:** Barbara Pucelik, Luis G. Arnaut, Janusz M. Dąbrowski

**Affiliations:** 1 Faculty of Chemistry, Jagiellonian University, 30-387 Kraków, Poland; barbara.pucelik@gmail.com; 2 Malopolska Centre of Biotechnology, Jagiellonian University, 30-387 Kraków, Poland; 3 CQC, Department of Chemistry, University of Coimbra, 3004-535 Coimbra, Portugal; lgarnaut@ci.uc.pt

**Keywords:** bacteriochlorins, chemokines, cytokines, photodynamic therapy, immune response

## Abstract

Photodynamic therapy (PDT) augments the host antitumor immune response, but the role of the PDT effect on the tumor microenvironment in dependence on the type of photosensitizer and/or therapeutic protocols has not been clearly elucidated. We employed three bacteriochlorins (F_2_BOH, F_2_BMet and Cl_2_BHep) of different polarity that absorb near-infrared light (NIR) and generated a large amount of reactive oxygen species (ROS) to compare the PDT efficacy after various drug-to-light intervals: 15 min. (V-PDT), 3h (E-PDT) and 72h (C-PDT). We also performed the analysis of the molecular mechanisms of PDT crucial for the generation of the long-lasting antitumor immune response. PDT-induced damage affected the integrity of the host tissue and developed acute (protocol-dependent) local inflammation, which in turn led to the infiltration of neutrophils and macrophages. In order to further confirm this hypothesis, a number of proteins in the plasma of PDT-treated mice were identified. Among a wide range of cytokines (IL-6, IL-10, IL-13, IL-15, TNF-α, GM-CSF), chemokines (KC, MCP-1, MIP1α, MIP1β, MIP2) and growth factors (VEGF) released after PDT, an important role was assigned to IL-6. PDT protocols optimized for studied bacteriochlorins led to a significant increase in the survival rate of BALB/c mice bearing CT26 tumors, but each photosensitizer (PS) was more or less potent, depending on the applied DLI (15 min, 3 h or 72 h). Hydrophilic (F_2_BOH) and amphiphilic (F_2_BMet) PSs were equally effective in V-PDT (>80 cure rate). F_2_BMet was the most efficient in E-PDT (DLI = 3h), leading to a cure of 65 % of the animals. Finally, the most powerful PS in the C-PDT (DLI = 72 h) regimen turned out to be the most hydrophobic compound (Cl_2_BHep), allowing 100 % of treated animals to be cured at a light dose of only 45 J/cm^2^.

## 1. Introduction

Photodynamic therapy (PDT) is a promising therapeutic approach to cancer, which in recent years entered the mainstream therapeutic options in oncology [1,2,3]. PDT aims at the destruction of the primary tumor without damaging surrounding, healthy tissues, and at the stimulation of anti-tumor immunity. There are three main mechanisms of PDT: the direct killing of cancer cells by photogenerated reactive oxygen species (ROS), tumor-associated vascular damage and activation of anti-tumor immune responses [2,4]. These mechanisms suggest that intricate relations between the nature of the photosensitizer (PS), PS dose, light dose, drug-to-light interval (DLI), the oxygen concentration in the targeted tissue and the mechanism of cell death [2,5,6,7] are likely to affect the outcome of PDT efficacy.

In practice, three fundamental PDT approaches are investigated: vascular-targeted PDT (V-PDT), tumor cellular-targeted PDT (C-PDT) and endothelial cells-targeted PDT (E-PDT). V-PDT is characterized by short drug-to-light intervals (DLI = 5–15 min). The main target of this protocol is the tumor vascularization. C-PDT targets tumour cells directly through specific molecules on cancer cells’ surfaces. In this case, longer drug-to-light intervals (DLI > 12h) are used [8,9,10,11]. At intermediate DLIs (e.g., 3 h) the PS is found mainly in the blood and endothelial cells, with partial accumulation in tumor cells [6,12], and protocols using such intermediate DLIs are designated here as E-PDT.

The first step in PDT is the selection of a PS with physicochemical and pharmacological properties adequate for the intended PDT regime [13]. The hydrophilicity/lipophilicity of the PS is one of the most important factors influencing its biological behavior, including uptake by cancer cells, intracellular localization, biodistribution and pharmacokinetics [14,15]. The high expression of low-density lipoprotein (LDL) receptors in tumors makes LDL-bounded lipophilic PSs more likely to achieve LDL-receptor-mediated endocytosis than hydrophilic drugs. These, in contrast, are mainly associated with serum proteins such as albumin and tend to localize in tumor vessels [16]. Many second-generation PSs were designed to be rather hydrophobic in order to increase their accumulation in tumors, and thus improve the outcome of C-PDT [17]. The challenge of aggregation of hydrophobic PS in biological fluids has been addressed using delivery vehicles such as liposomes, lipoproteins or micelles. Hydrophilic photosensitizers are much easier to prepare for *in vivo* administration, but they are less likely to permeate cancer cells and tend to show faster clearance from the organism. Nevertheless, their tendency to interact with plasma proteins following intravenous administration (*i.v.*) [18] can lead to significant vascular effects in V-PDT [19,20]. Amphiphilic compounds, which contain both hydrophilic and hydrophobic moieties in their molecular structure, are quite versatile and promising for clinical translation [21,22,23]. Their pharmacokinetics and biodistribution largely determine the choice of the optimal drug-to-light interval [15,24], and the possibility to achieve PS accumulation in both individual cell organelles and intracellular spaces [25].

Photosensitizers bearing fluorine, chlorine or other halogen atoms usually display an enhanced photodynamic effect [26,27,28]. This is assigned to the so-called “heavy atom effect” resulting from the increased spin-orbit coupling originating from atoms with higher atomic numbers, and consequently, higher spin-orbit coupling constants (ζ = 0.24 for H, ζ = 269 for F and ζ = 586 for Cl). Hence, the internal heavy atom effect accelerates the S_1_→T_1_ intersystem-crossing rate, increases the triplet quantum yields (Φ_T_) of photosensitizers and consequently improves the ability to generate ROSs. In addition, the introduction of fluorine and chlorine atoms into the PS structure leads to a change in their polarity, which in turn can increase the rate of transport of biologically-active compounds through lipid membranes [29,30,31,32,33,34].

It is widely recognized that PDT regimes can trigger rapid inflammatory responses that are essential for the activation of antitumor immunity [35,36,37], improving long-term tumor control and offering protection against metastasis [38]. Unlike most chemotherapies, which mainly lead to apoptotic cell death, PDT may also lead to necrosis or immunogenic cell death (ICD), which can result in controlled inflammation, which in turn leads to further stimulation of the immune system [7,37,39,40,41]. The generation of the post-PDT antitumor response involves the participation of various mechanisms based on the secretion of cytokines and mediators associated with the inflammatory process, increased level of neutrophils in the blood combined with neutrophil recruitment to the treated areas, as well as induction of acute-phase proteins and activation of the complement system [7,35,40,42]. PDT-induced inflammation is associated with the influx of the cells of the immune system to the treated area, and these cells contribute to the development of an immune response that recognizes cells that survived the therapy or are found in other parts of the body in the form of metastases [21]. 

Changes in the peritumoral microenvironment induced by PDT seem to influence the activity of non-specific mechanisms of the innate immune system. The cells involved in non-specific mechanisms include phagocytic cells—macrophages, granulocytes, monocytes and several mediators (chemokines and cytokines) [43,44]. PDT-induced inflammation may also activate mechanisms of adaptive (or specific) immune response generated when antigens are recognized by the lymphocyte population, capable of inducing immunological memory and of causing the regression of distant established tumors that received no treatment [17]. Cells dying after PDT can be a source of antigens and stimulate cells of the immune system that infiltrate the tumor, eventually leading to anti-tumor immunity [45,46,47]. The development of effective, adaptive immune responses requires the prior engagement of innate immunity [39].

**Scheme 1:**
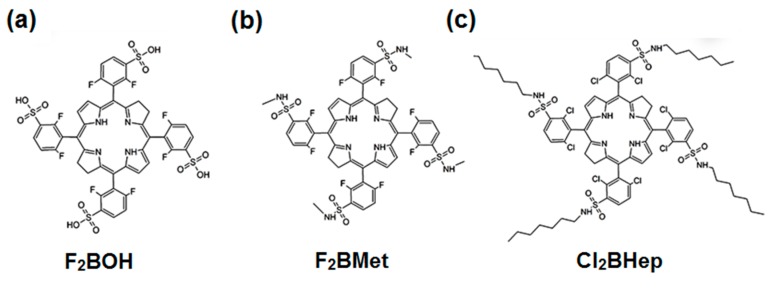
Chemical structure of investigated halogenated bacteriochlorin derivatives.

The main purpose of this work is to investigate PDT regimes with three bacteriochlorin-type photosensitizers of different polarity and to relate the outcomes of these regimes to the properties of the PSs, namely their physicochemical properties, subcellular localization, pharmacokinetics, ROS generation and immune response. This work offers a basis for the optimization of immune responses with PDT and the rational design of combinations between PDT and immunotherapies. The bacteriochlorins selected for this study are presented in Scheme 1. The synthesis and some photophysical properties of F_2_BOH, F_2_BMet (named redaporfin) and Cl_2_BHep have been reported [21,22,23,48], as well as the pharmacokinetics and biodistribution of F_2_BOH and F_2_BMet [14,15,24]. Although some *in vitro* studies and the PDT of tumor-bearing mice have been published for F_2_BOH and F_2_BMet [21,22,23,24], they were repeated and extended in this work to afford a more direct comparison with the corresponding studies with Cl_2_BHep.

## 2. Experimental Section

### 2.1. Chemicals and Photosensitizers

All commercial chemicals and reagents were of analytical grade and were purchased from Sigma-Aldrich. 5,10,15,20-tetrakis(2,6-difluoro-3-sulfophenyl)bacteriochlorin (F_2_BOH), 5,10,15,20-tetrakis(2,6-difluoro-3-N-methylsulfamoylphenyl)bacteriochlorin (F_2_BMet, redaporfin) and 5,10,15,20-tetrakis(2,6-dichloro-3-N-heptylsulfamoylphenyl)bacteriochlorin (Cl_2_BHep) were prepared according to reported procedures [21,22,23,48].

### 2.2. Spectroscopic Studies

Electronic absorption spectra were recorded in a Hewlett Packard HP8453 spectrophotometer. Solutions containing samples of photosensitizers were dissolved in the selected solvents in quartz cuvettes. Using measured absorbance for various concentrations of bacteriochlorins, either in dimethyl sulfoxide (DMSO) or in ethanol, the molar absorption coefficients were determined from Beer’s law. Fluorescence emission spectra were recorded from 700 nm to 800 nm with excitation at λ ≈ 505 nm. The excitation and emission slits were both set to 8 nm and scanning speed to 50 nm/min. Fluorescence spectra were recorded with a Perkin Elmer Fluorescence Spectrometer LS 55 (Perkin Elmer, Waltham, MA, USA). Fluorescence lifetimes were determined using a Time-Correlated Single Photon Counting (TCSPC) mode using the FluoroLog-3 Spectrophotometer (Horiba Jobin Yvon, Glasgow, UK). The instrument was equipped with 340 nm picoseconds-pulsed light-emitting diodes (LEDs) as the excitation source in the MCS mode. During measurements, the Instrument Response Function (IRF) was obtained from a non-fluorescence suspension of colloidal silica (LUDOX 30%, Sigma Aldrich, Schnelldorf, Germany), held in a 10 mm path length quartz cell, and was considered to be wavelength independent. All lifetimes were fit to a χ^2^ value of less than 1.1, and with residuals trace symmetrically distributed around the zero axes. Fluorescence quantum yield for F_2_BOH and Cl_2_BHep in DMSO was determined using the comparative method according to the Equation (1):

(1) ΦF= ΦFStdF·AStd·n2 FStd·A·nStd2,

where F and F_Std_ are the areas under the fluorescence emission curves of the sample and the standard, A and A_Std_ are the absorbance of the sample and the standard, and η/ηStd are the refractive indices of the solvent used for the sample and the standard, respectively. Redaporfin (Φ_FStd_ = 0.138) was employed as the standard. The absorbance of the solutions at the excitation wavelength was in the range of 0.02.

### 2.3. ROS Detection with Fluorescent Probes

3’-p-(aminophenyl)fluorescein (APF) and 3’-p-(hydroxyphenyl)fluorescein (HPF) were used to detect hydroxyl radicals. Singlet Oxygen Sensor Green® (SOSG) was applied as a probe for singlet oxygen. Dihydroethidinum (DHE) was used to identify the superoxide ion. All probes were employed for the detection of various ROSs after the illumination of the photosensitizer. PS solutions were diluted to a final concentration of 5 μM per well. A tested fluorescent probe was added at a final concentration of 10 μM. PS solutions were irradiated for various time intervals with a red light from the LED-based device (735 ± 20 nm). A microplate reader (Tecan, Infinite M200 Reader, Tecan, Männedorf, Switzerland) was used for the acquisition of fluorescence signal immediately before and after illumination. APF fluorescence emission at 515 nm was determined upon excitation at 490 nm. For SOSG, the corresponding values were 525 and 505 nm, and 480 nm and 580 nm for DHE.

### 2.4. Photodecomposition Experiments

The photostability of bacteriochlorins was investigated following the measurement of their optical absorption in 5 μM solutions prepared in DMSO and diluted in phosphate-buffered saline (PBS) (the DMSO content did not exceed 0.5%). Continuous irradiation of an aerated solution was carried out using a LED at 735 ± 20 nm. The reaction progress was monitored by electronic spectroscopy using a Hewlett Packard HP8453 spectrophotometer.

### 2.5. LogP Determination

The n-octanol/PBS partition coefficients (logP) were determined following the shake-flask method. The photosensitizers were dissolved in the mixture of n-octanol previously saturated with a solution of PBS and the same volume of PBS saturated with n-octanol. After the addition of the samples of each bacteriochlorin, the solutions were mixed on a vortex device (horizontally) and then the phases were separated by centrifugation (15 min, 8000 rpm, RT). Next, the PBS/n-octanol phase was taken and diluted to obtain 0.5% of PBS/n-octanol content in the final solution. This solution was left into the ultrasonic bath. The fluorescence of each solution was measured using a Fluorescence Spectrometer LS 55 (Perkin Elmer, Waltham, MA, USA) and compared with the calibration curve to obtain the concentration of the photosensitizer. Partition coefficients were calculated from the ratio c_oct_/c_PBS_, where c_oct_ and c_PBS_ are the concentrations of the porphyrin derivatives in the n-octanol and in the PBS.

### 2.6. In Vitro Studies

#### 2.6.1. Photosensitizer Formulation

For the biological studies, due to the reduced solubility of Cl_2_BHep in water, the Pluronic-based micelles were applied to deliver it either to cells *in vitro* or to tumors *in vivo*. The formulation was prepared by a thin-film hydration method. Dynamic Light Scattering (DLS) using a Malvern Zetaziser Nano ZS system was employed to measure the hydrodynamic diameters (D_H_), size distributions and zeta potentials (ζ) of polymeric micelles. Cl_2_BHep concentrations in the formulations were determined and controlled by UV−vis spectrophotometry. Results are reported as the mean of three independent measurements with three different samples (*n* = 3). The weight percentage of Pluronic-based formulations was determined spectrophotometrically using the absorption calibration curves generated from the Cl_2_BHep standard solutions at known concentrations, as well as DLS measurements. Drug loading content (DL) and drug encapsulating efficiency (EE) were calculated based on the Equations (2) and (3) below [12]:

(2) $$DL\left(\%\right)=\frac{weight\;of\;PS\;in\;micelles}{weight\;of\;micelles}\cdot 100\%\;$$

(3) $$EE\left(\%\right)=\frac{weight\;of\;PS\;in\;micelles}{weight\;of\;the\;feeding\;PS}\cdot 100\%$$

Electronic absorption spectra were recorded by HP8453 spectrophotometer using a 1 cm path-length quartz cell.

#### 2.6.2. Cell Cultures

Murine colon carcinoma (CT26) cells were grown in full-strength Dulbecco’s modified Eagle’s medium (DMEM) with 4.5 g/L glucose, L-glutamine, sodium pyruvate and 3.7 g/L NaHCO_3_ (BioTech) with the addition of 10% fetal bovine serum (FBS, PAN-Biotech, Aidenbach, Germany) and supplemented by antibiotics (penicillin/streptomycin, PAN-Biotech, Aidenbach, Germany). The cells were cultured in incubators maintained at 37 °C with 5% CO_2_ under fully humidified conditions. All experiments were performed on cells in the logarithmic phase of growth. Media were replaced every two days and cells were subcultured using 0.25% trypsin-ethylenediaminetetraacetic acid (EDTA). 

#### 2.6.3. Cellular Uptake

CT26 cells were seeded on 96-plate microplate (10^4^ per well). After 24 h, the cells were incubated with various PS concentrations for time intervals from 2 h up to 24 h. Bacteriochlorin solutions were prepared by diluting stock solutions in DMSO or in micellar formulations (for Cl_2_BHep) with the culture medium to the desired final concentration (5 μM). The highest concentration of DMSO in the medium did not exceed 0.5%. After incubation, the cells were washed twice with PBS and solubilized in 30 μL of Triton X-100 and 70 μL of DMSO/ethanol solution (1:3). The retention of cell-associated PSs was detected by fluorescence (λ_exc_ = 505, λ_em_ = 750 nm) with the microplate reader (Tecan Infinite M200 Reader, Tecan, Männedorf, Switzerland). 

#### 2.6.4. Cytotoxicity and Cell Survival Assay

The MTT (3-(4,5dimethylthiazol-2-yl)2,5-diphenyl tetrazolium bromide) assay was used to quantify cell survival and PS-mediated cytotoxicity. After the cells were attached to the microplates, PS solutations at concentrations of 0 to 100 μM were added to the cell medium. Cells were incubated for the appropriate time in the dark. Next, the PS solution of each well was removed, cells were washed in PBS and fresh culture medium supplemented with FBS and antibiotics was added to each well, and cells were returned to the incubator for 24 h. MTT dissolved in PBS at content 10% of final solution was added to each well, and the microplates were further incubated for ca. 3 h. Then, the medium was discarded, and 100 μL of the mixture of DMSO:methanol (1:1) were added to the cultures and mixed thoroughly to dissolve the dark blue crystals of formazan. 

Formazan quantification was performed using an automatic microplate reader (Tecan Infinite M200 Reader) by absorbance measurements at a 565 nm. Each essay was replicated in at least three independent experiments.

#### 2.6.5. Photodynamic Effect

On the basis of the cytotoxicity results, a nontoxic concentration of bacteriochlorin (5 μM) was selected. Cells were incubated for the optimal time determined experimentally in the dark with each bacteriochlorin solution in the culture medium. After this incubation time, the cells were washed with PBS and irradiated with a 735 ± 20 nm LED irradiation system. An LED illuminator (Photon Institute, Kraków, Poland) and sample chamber with stabilized temperature and the display was employed for illumination of the plate with seeded cells. The diode (735 nm ± 20 nm) was adjusted to give a uniform spot of irradiated area with a fluence rate of 1.072 mW as measured with a power meter (Light Engine UNO 50, Tampa, FL, USA), and to excite the same number of molecules. Next, the cells were washed with fresh medium and the plates were returned to the incubator for 24 h. Cell viability was determined by MTT assay in independent experiments performed 24 h post-irradiation. Each essay was replicated at least in three independent experiments. 

#### 2.6.6. Intracellular Localization—CLSM Imaging

The intracellular accumulation of bacteriochlorins was assessed in CT26 cancer cells. Prior to imaging, CT26 cells were seeded on microscopic slides at a density of 1·10^5^ cells, and were kept at 37 °C in a 95% air- and 5% CO_2_-humidified atmosphere, for 24 h. After being washed with fresh medium, the cells were incubated in the dark with 10 μM solution of each bacteriochlorin-prepared cell medium, for 24 h. Next, after being washed with Hank’s Balanced Salt Solution (HBSS, composition: 0.14 M NaCl, 0.005 M KCl, 0.001 M CaCl_2_, 0.0004 M MgSO_4_·7H_2_O, 0.0005 M MgCl_2_·6H_2_O, 0.0003 M Na_2_HPO_4_·2H_2_O, 0.0004 M KH_2_PO_4_, 0.006 M D-glucose, 0.004 M NaHCO_3_), the cells were incubated with specific intracellular organelle probes: 100 nM Mito-Tracker green, 1 µM ER-Tracker green, 1μM Lyso-Tracker red (Molecular Probes, Invitrogen Life Technologies, CA, USA), diluted in HBSS buffer. In addition, cells were incubated for 10 min with Hoechst33342. After 30 min incubation, at 37 °C, in the dark, the cells were washed with HBSS two times, and the slide was transferred to the microscope stage, and cells were visualized under a confocal microscope Zeiss LSM 880 (Carl Zeiss, Jena, Germany) with a 40× oil immersion objective. Images were analyzed by Zeiss ZEN Software.

### 2.7. In Vivo Studies

#### 2.7.1. Tissue Distribution and Pharmacokinetics of Cl_2_BHep in BALB/c mice 

All experiments were carried out with approval no. 42/2014 and 190/2018 of the First Ethics Committee for Research on Animals, Kraków, Poland. BALB/c (8−9-weeks-old males, Animal Facility, FBBiB JU) were used to perform in vivo studies. The animals were humanely treated and supplied with food and water ad libitum. The CT26 cells (0.5 × 10^6^ in PBS suspension) were implanted subcutaneously into the right thigh of male BALB/c mice. The intravenous administration of the drug formulation was done when the tumor attained a diameter of 5−6 mm in each animal, which usually took about seven days after inoculation. Each formulation, corresponding to 1.5 mg·kg^−1^ of Cl_2_BHep was injected into the tail vein of each animal, and tissue distribution was evaluated at 15 min, 3 h and 72 h after administration. At the selected time points post-injection, the mice were anesthetized with ketamine and xylazine and sacrificed. For each animal, selected organs and tissue samples were collected separately and weighed. To extract the pigments, we separately homogenized tissue samples in EtOH/DMSO solution (75:25) over 1 min using a Yellowline by an IKA DI 25 basic animal tissue homogenizer. The homogenate was centrifuged, the supernatant was collected, and the pellet was re-extracted four more times using the procedure described above to ensure complete recovery of the drug. The extracts were pooled and the fluorescence analysis of the extracts was done. The samples were excited at 505 nm, and the fluorescence spectra were recorded in a range of 600−800 nm. The amount of Cl_2_BHep in the tissues was reported as the average from four animals (with the standard error of the mean (SEM)). The concentration of Cl_2_BHep was estimated from the fluorescence intensity of this bacteriochlorin in samples with known concentrations.

#### 2.7.2. *In Vivo* Photodynamic Therapy with Bacteriochlorins 

The antitumor efficacy of the bacteriochlorins was evaluated in BALB/c mice bearing CT26 tumors. When the tumor volume reached about 4−5 cm in diameter, these mice were randomly assigned to experimental groups (*n* = 6). For safe PS administration, due to the lipophilic character of each compound, the F_2_BOH was prepared in saline solution, F_2_BMet was formulated in CrEL/EtOH/NaCl 0.9% (0.2:1:98.8, *v*:*v*:*v*)^22^, and Cl_2_BHep was encapsulated in Pluronic P123 (10% *w*/*v*) micelles^12^. Each bacteriochlorin was administered via the tail vein injection with a dosage of 1.5 mg kg^−1^ BW. The tumor irradiation was performed at DLI = 15 min, DLI = 3 h or DLI = 72 h using a laser (Omicron laser model LDM750.300.CWA.L.M equipped with optical fiber model FD/Medlight, Ecublens, Switzerland) at 750 nm, radiant exposures ranging 45−105 J cm^−2^ and a laser power of 130 mW. The illuminated area with a diameter of 1.3 cm was kept constant during irradiation. Survival curves were estimated by means of the Kaplan−Meier analysis. 

#### 2.7.3. Analysis of Serum Inflammatory Cytokines with MAGPIX System

To determine the concentration of a panel of inflammatory cytokines (IL-6, IL-10, IL-13, IL-15, KC, MIP-2, LIX, LIF, MCP-1, MIP-1α, MIP-1β, IP-10, MIG, GM-CSF, TNFα and VEGF) listed in Table 1, in the mice sera (*n* = 6–7) before and after treatment, the MAGPIX system was employed. The test was replicated three times.

**Scheme 2:**
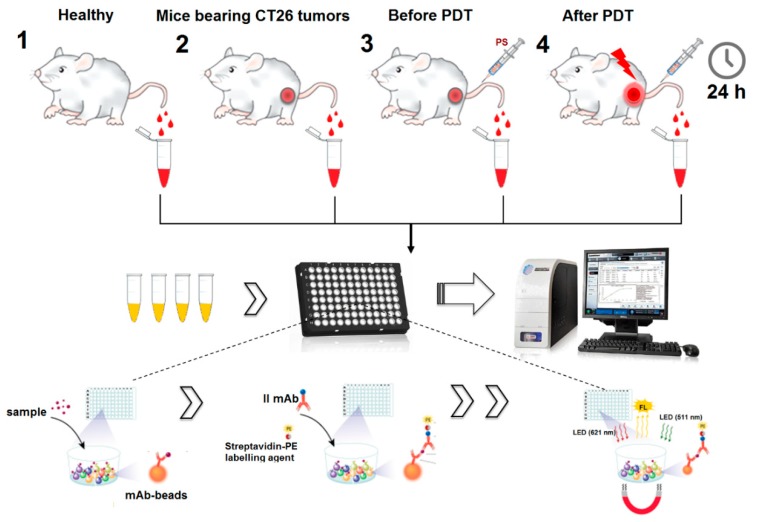
Scheme for carrying out immunological analyses using the MAGPIX system.

The analysis was performed using the MCYTOMAG-70K 16-Plex Panel (MerckMillopore, Burlington, MA, USA) according to the manufacturer’s instructions. In total, samples from healthy and tumor-bearing mice, as well as before and after treatment, were tested (Scheme 2). The results were analyzed with the MAGPIX xPONENT software (Burlington, MA, USA). 

### 2.8. Statistical Analysis

In both physicochemical and photochemical studies, as well as *in vitro* biological activity evaluation, the results are expressed as an average ± the standard error of the mean (SEM). The sample size in biological tests *in vitro* was *n* = 6–12 in each experimental group. All experiments were repeated independently at least three times. The Student’s t-test or Welch’s Mann–Whitney U-test (Luminex analysis) was used for the determination of statistical significance. Results were considered as statistically significant with a confidence level of 95% (*p* < 0.05). Statistical analysis was performed with the STATISTICA 12.5 (StatSoft, Kraków, Poland).

## 3. Results

### 3.1. Photosensitizers

The synthesis and characterization of F_2_BOH, F_2_BMet and Cl_2_BHep from their halogenated 5,10,15,20-tetraphenylporphyrin precursors were recently described elsewhere [23,27,48].

### 3.2. Spectroscopic Characterization of Photosensitizers

**Figure 1:**
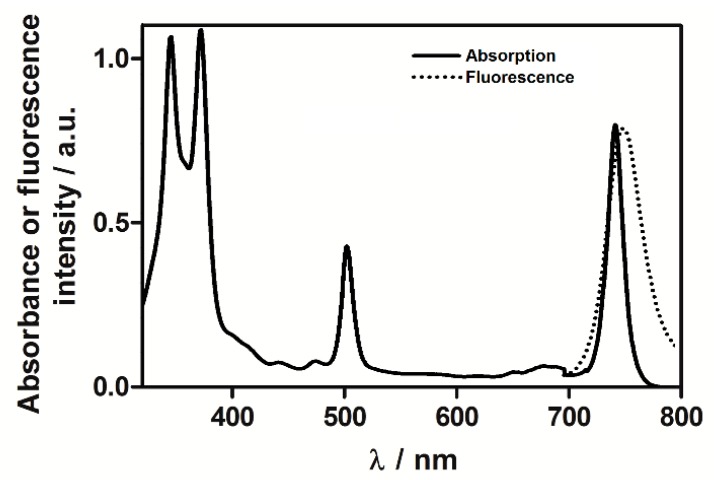
Representative, normalized absorption and fluorescence spectra of F_2_BMet in dimethyl sulfoxide (DMSO).

All investigated photosensitizers exhibit the characteristic bacteriochlorin absorption spectrum with a broad near-UV Soret, a narrow band in the green (≈ 510 nm) and a near-infrared Q_y_ band (≈ 745 nm) of high intensity that is crucial for PDT. Representative absorption and fluorescence spectra recorded in DMSO are shown in Figure 1. Table 2 summarizes the important photophysical properties of these photosensitizers. The high molar absorption coefficients of these photosensitizers enable their use at much lower concentrations than their porphyrin and chlorin analogs [5]. Moreover, their absorption of NIR light allows for the use of light that penetrates more deeply into human tissues than red light.

The fluorescence lifetimes of F_2_BOH and F_2_BMet are comparable. This is due to their structural similarity and the presence of eight fluorine atoms in the phenyl rings located in the *meso* positions of the tetrapyrrole macrocycle. Cl_2_BHep is characterized by a much shorter fluorescence lifetime, as expected from the presence of eight chlorine atoms and their higher spin–orbital coupling constants (ζ) than fluorine atoms. The difference in fluorescence quantum yields can be assigned to the same effect. Cl_2_BHep indicates the lowest Φ_F_ value among other tested compounds (Φ_F_ = 0.008). The relatively high value of Φ_F_ determined for F_2_MBet suggests the use of this PS in photodiagnosis. 

### 3.3. ROS Detection by Fluorescent Probes

**Figure 2:**
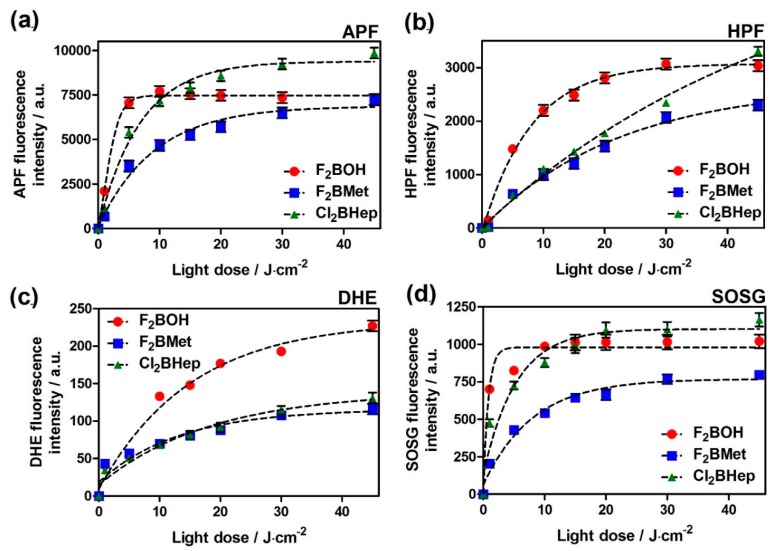
Generation of reactive oxygen species (ROSs) in bacteriochlorin solution (in phosphate-buffered saline (PBS), dimethyl sulfoxide (DMSO) did not exceed 0.5%) assessed by the fluorescence probes selective towards: (**a**) overall radical species (APF), (**b**) hydroxyl radicals (HPF), (**c**) superoxide radical anion (DHE) or (**d**) SOSG specific for singlet oxygen. The concentrations of the probes were initially 10 μM, and bacteriochlorin was 5 μM in each sample. Samples were irradiated with a 735 ± 20 nm LED light. Data are presented as mean ± the standard error of the mean (SEM) (*N* = 12).

The ability of bacteriochlorins to produce different ROSs after illumination was monitored using APF, HPF, DHE and SOSG fluorescent probes. Figure 2 shows that all investigated bacteriochlorins activate probes specific for singlet oxygen as well as for oxygen-centered radicals. F_2_BOH and F_2_BMet tend to give lower SOSG fluorescence intensities than Cl_2_BHep (Figure 2d), which is consistent with the higher singlet oxygen quantum yield of Cl_2_BHep presented in Table 2. Cl_2_BHep also tends to give higher APF and HPF fluorescences than other bacteriochlorins, suggesting that this derivative is the most effective in producing oxygen-centered radicals (Figure 2a,b). This is consistent with determinations of the triplet quantum yields of chlorinated and fluorinated tetraphenyl-bacteriochlorin derivatives which show that the triplet quantum yields of chlorinated derivatives reach unity, whereas those of the fluorinated compounds do not seem to exceed 0.8 [21,22,23]. All bacteriochlorins studied here activate dihydroethidium (DHE), thereby indicating the presence of a superoxide anion, as shown in Figure 2c. F_2_BOH led to higher activations than F_2_BMet and Cl_2_BHep. It will be shown below that, under the same condition, F_2_BOH is substantially decomposed. It is possible that this decomposition affects the probe. The balance between type I (hydroxyl radicals) and type II (singlet oxygen) photochemical mechanisms is thought to play an important role in the PDT activity because the hydroxyl radicals are the most cytotoxic species, and are expected to be a major contributor to tumor damage in PDT. Tookad Soluble [50], which is also a bacteriochlorin derivative, acts mainly via type I photochemistry in aqueous media [51,52]. This PS forms both superoxide and hydroxyl radicals upon illumination and the reaction of superoxide with nitric oxide in tumor blood vessels was proposed as the major factor related to the antivascular effect induced by Tookad-V-PDT [51,53]. The propensity of halogenated bacteriochlorins to produce ROS via a type I mechanism was attributed to the formation of a charge-transfer complex with molecular oxygen [3].

### 3.4. Photostability 

**Figure 3:**
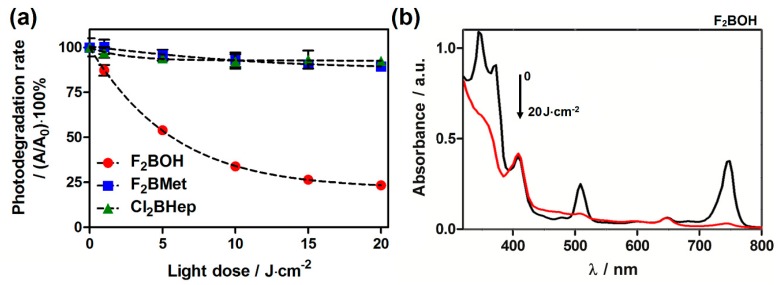
Photostability of sulfonated and sulfonamide bacteriochlorins in PBS solutions (<0.5% DMSO) under a near-infrared (NIR) light-emitting diode (LED) irradiation: (**a**) the photodegradation rate; (**b**) the changes in absorption spectra of F_2_BOH during irradiation.

The photodecomposition quantum yield Φ_pb_ is a measure of photostability. According to Table 2, F_2_BOH is the least stable bacteriochlorin of this series. This can also be appreciated in Figure 3. It was shown that bacteriochlorins with phenylsulfonic groups have lower oxidation potentials than bacteriochlorins with phenylsulfonamide groups, and this is associated with more facile oxidation [21,22,23]. Figure 3b shows that the disappearance of the bacteriochorin bands at 375, 510 and 743 nm is not accompanied by corresponding changes in the shoulder at 410 nm or in the small band at 660 nm, both assigned to small chlorin contamination in this sample. This suggests that the photodecomposition of F_2_BOH will eventually lead to the opening of the macrocycle. This photodecomposition is likely triggered by the ROS produced by F_2_BOH, and may lead to other radical intermediates that react with fluorescent probes, namely DHE. In contrast, the sulfonamide bacteriochlorins are remarkably stable.

### 3.5. Lipophilicity as Assessed by logP Determinations

n-Octanol:PBS partition coefficients are a convenient measure of polarity and are related to solubility in biocompatible solvents. The *P*_OW_ values determined using the shake-flask method are presented in Table 2. The obtained logP values are similar to those previously reported [48]. The log*P* values range from −1.4 to 4.5, meaning that F_2_BOH is soluble in water, but Cl_2_BHep is insoluble in water. Fluorine and chlorine atoms present in the *ortho*-position of the phenyl group have a similar effect on the photostability of the studied compounds. On the other hand, substituents in the *meta*-position determine their hydrophilicity. Therefore, the most hydrophobic compound is a derivative containing *n*-heptyl chain-Cl_2_BHep. Moreover, such large differences in polarity are expected to lead to very different biological effects [54]. 

### 3.6. Biological Studies—Optimization of PS Formulation

**Figure 4:**
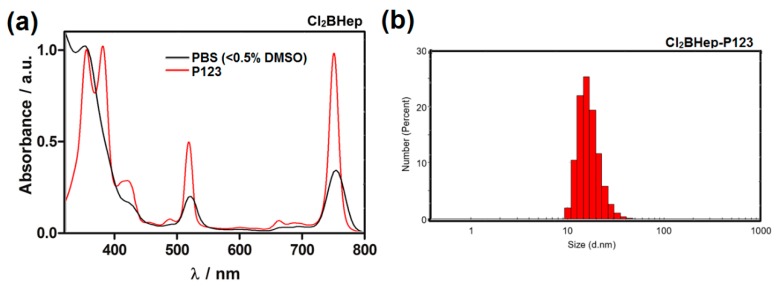
The impact of Cl_2_BHep encapsulation in P123 micelles: (**a**) Absorption spectra of Cl_2_BHep registered in PBS (< 0.5% DMSO) and micellar solution in PBS after its encapsulation in Pluronic P123; (**b**) particle size distribution of Cl_2_BHep-P123 measured by dynamic light scattering (DLS).

Formulations of F_2_BMet in Pluronic poloxamers were recently described [12] and motivated the development of similar formulations fo Cl_2_BHep. Absorption spectra of Cl_2_BHep in PBS with < 0.5% DMSO and encapsulated in polymer micelles are shown in Figure 4. The encapsulation of this bacteriochlorin derivative significantly increases the sharpness of the bands, suggesting disaggregation in P123 micelles. Particle size distribution estimated by DLS (Figure 4b) shows that the hydrodynamic diameter of Cl_2_BHep-P123 micelles prepared in PBS at pH 7.4 is ca. 27 nm with a relatively narrow and homogeneous size distribution. Similarly to P123 micelles prepared for F_2_BMet described previously [12], the calculated (see equations above) encapsulation efficacy (EE) reached ca. 87% with 12.12 (wt %) drug loading content (DL). The zeta potential for Cl_2_BHep was slightly negative and reached ζ = −1.07. These data indicate that P123 gave the stable structures, with the ability to form micelles and to provide a slightly hydrophobic environment to maintain the photosensitizer in the monomeric form [12]. P123 micelles may increase cellular uptake, efficient photogeneration of hydroxyl radicals, as well as preferable tissue distribution and pharmacokinetics [12].

### 3.7. Cellular Uptake of Bacteriochlorins 

**Figure 5:**
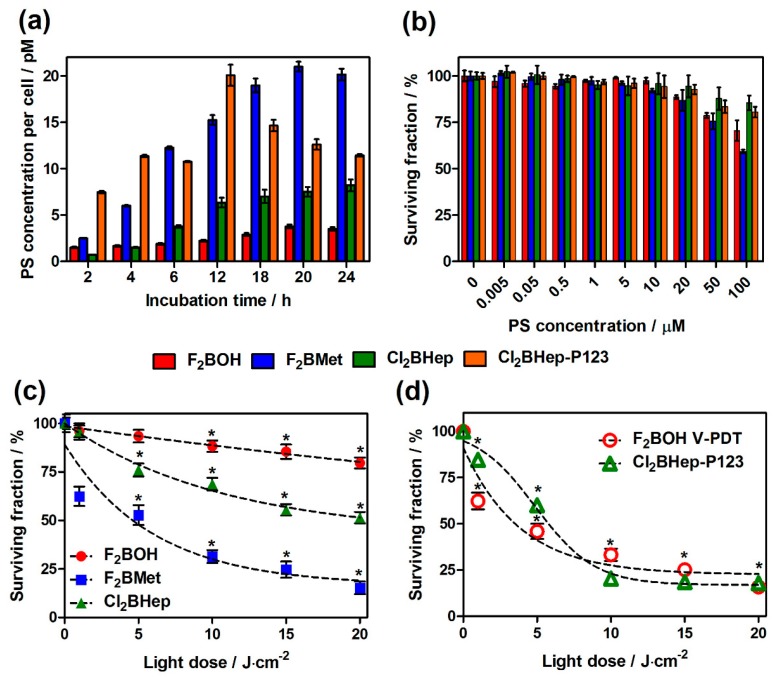
*In vitro* activity of investigated photosensitizers: (**a**) time-dependent uptake of halogenated bacteriochlorins at 5 μM by CT26 cells determined based on fluorescence measurements of cellular extracts; (**b**) cytotoxicity in the dark toward CT26 cells determined by MTT assay after optimal incubation time estimated in uptake studies; (**c**) photodynamic efficacy of F_2_BOH, F_2_BMet and Cl_2_BHep after their incubation at 5 μM with CT26 for optimal time and irradiation with 735 ± 20 nm LED light; (**d**) phototoxicity of F_2_BOH at 5 μM without washing the medium before illumination, and of Cl_2_BHep encapsulated in P123 micelles at 5 μM. Data are presented as mean ± SEM (*N* = 12), * *p* < 0.05.

The time-dependent accumulation of F_2_BOH, F_2_BMet and Cl_2_BHep (in PBS and in P123 micelles) in CT26 cells exposed to a 5 μM photosensitizer solution are shown in Figure 5a.

The results show that for all tested compounds, a gradual, time-dependent increase in PS accumulation was observed. The optimal accumulation for each bacteriochlorin ranged from 18 to 24 h, and then the uptake decreases, or a plateau phase is observed. F_2_BMet (log *P* = 1.9) showed the most effective accumulation in cells at a concentration of 24 pM per cell after 20 h of incubation. F_2_BOH is deprotonated in the culture medium (log *P* = −1.4), and its negative charges limit its ability to penetrate cell membranes, leading to a relatively low accumulation in cells (3.88 pM per cell). For this type of compound, penetration into cells is mainly through the endocytosis. Cl_2_BHep (log *P* ≈ 4.5) is likely to aggregate in the medium which limits its bioavailability and justifies its low concentration in cells (ca. 7.5 pM). The encapsulation of Cl_2_BHep in P123 micelles improves the cellular uptake (22 pM), especially at shorter incubation times (12 h).

The results also indicate very low cytotoxic behavior in the dark in the whole wide range of PS concentrations investigated (Figure 5b) and pronounced phototoxicity especially for F_2_BMet (Figure 5c). Based on these data, it can be concluded that without the use of micelles, the largest photodynamic effect was noted for F_2_BMet, followed by Cl_2_BHep and finally by F_2_BOH. This is also the order of their uptake (Figure 5a). The lower uptake and lower phototoxicity of F_2_BOH can be overcome by illuminating the cells after the incubation with this photosensitizer, but without washing the photosensitizer molecules that remain in the culture medium. This procedure has a close analogy with V-PDT, where the PS is present in the blood, but not inside the cells. The results obtained in these conditions are presented in Figure 5d as F_2_BOH V-PDT. The possible aggregation of Cl_2_BHep can be avoided using Pluronic formulations. Figure 5d also shows that a P123 formulation of Cl_2_BHep significantly increases its phototoxicity without increasing the cytotoxicity in the dark (Figure 5b) The lethal doses of light causing the killing of 50% of the cancer cells population (LLD_50_) of the three photosensitizers are presented in Table 3.

### 3.8. Intracellular Localization—CLSM Imaging

**Figure 6:**
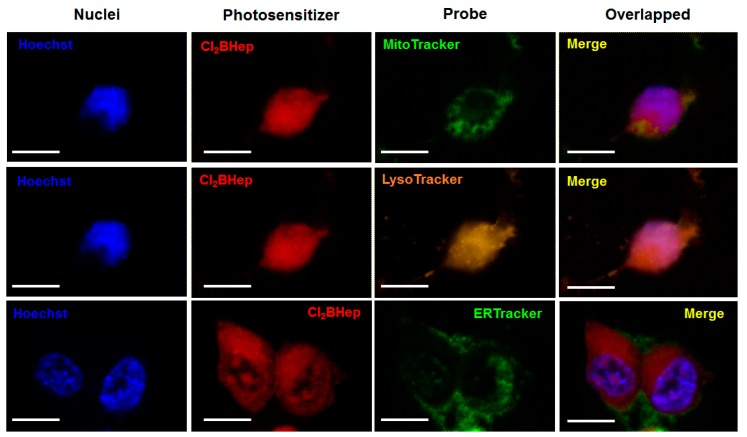
Confocal images of CT26 cells showing intracellular localization of Cl_2_BHep delivered in P123 micelles. Cells were marked with specific probes for mitochondria (Mito-Tracker), lysosomes (Lyso-Tracker) and endoplasmic reticulum (ER-Tracker).

The intracellular localization in CT26 cells of F_2_BOH, F_2_BMet and Cl_2_BHep was investigated by confocal laser scanning microscopy (CLSM). Each of these photosensitizers was incubated with a specific probe for mitochondria (MitoTracker), lysosomes (LysoTracker) and endoplasmic reticulum (ER-Tracker). The intracellular localization of F_2_BOH and F_2_BMet in different cell lines has been published before [21,22,23,41]. The more hydrophilic bacteriochlorin is accumulated in lysosomes, whereas the amphiphilic bacteriochlorin is mostly found in the ER (and Golgi), with a smaller localization in the mitochondria. The overlapped images corresponding to Cl_2_BHep are shown in Figure 6, in which the fluorescence of the bacteriochlorin is shown in red, and the fluorescence of the organelle-specific probe is shown in green (ER, mitochondria) or in yellow for lysosomes, respectively. The nuclei were also stained with Hoechst33342 and indicate the characteristic blue fluorescence. The lipophilic Cl_2_BHep possesses a high degree of localization in mitochondria and lysosomes, and a slightly lower one in the ER. The higher subcellular localization of sulfonamide bacteriochlorins in the ER and mitochondria is consistent with their higher photoxicities than F_2_BOH. It is worth to notice that the localization of a photosensitizer is important to determine the initial targets of PDT [18,55,56]. 

**Figure 7:**
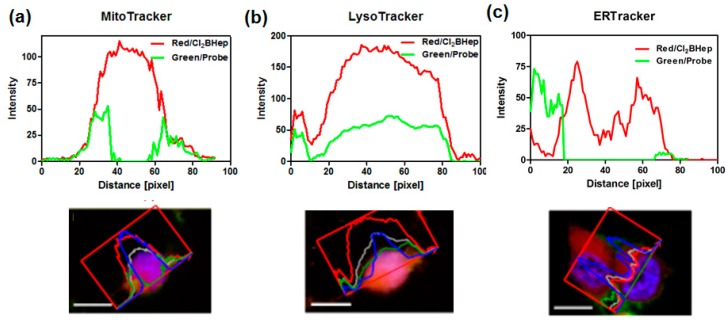
Colocalization of the photosensitizer (PS) and organelle-specific probes (**a**) MitoTracker, (**b**) Lysotracker and (**c**) ERTracker determined and quantified based on the confocal microscopy images and analyzed by ZenSoftware (Zeiss, Jena, Germany).

Furthermore, to confirm the colocalization of Cl_2_BHep (red signal) with each organelle-specific probe (green signal) more clearly, the spectral profiles for these components were defined. The obtained profiles are presented in Figure 7.

Localization of hydrophobic PSs in mitochondria may result from the influence of mitochondrial membrane potential and lipid bilayer membrane. Moreover, P123 strongly attenuated the uptake and photocytotoxicity of hydrophobic PSs and redirected the cellular uptake to endocytosis. For example, thiamine pyrophosphate (ThPP) solubilized with P123 was found to accumulate in endocytic vesicles, indicating endocytosis as the main mechanism for the cellular uptake of P123-solubilized THPP. Berg and co-workers found that the hydrophobic 5,10,15,20-tetrakis(4-hydroxyohenyl)porphine solubilized with P123 accumulated in endocytic vesicles, and suggested that in this case, the endocytosis is the main mechanism for cellular uptake [57]. The accumulation of the photosensitizer in certain specific organelles (mitochondria, endoplasmic reticulum or Golgi) is thought to lead to the more efficient triggering of cell death upon illumination [17,18,56].

### 3.9. In Vivo Studies: PDT Protocol Optimization—Biodistribution

Biodistribution and pharmacokinetic studies of *is done in* [24] and amphiphilic F_2_BMet [15,22] were reported recently, and reveal the highest tumor-to-muscle or -to-skin ratio immediately after PS intravenous administration [15,22,24]. F_2_BOH is characterized by high blood concentration after intravenous administration, relatively low tissue accumulation and rapid elimination time (44 h). Its *i.v.* injection does not require the use of any carriers and is done in PBS [24]. On the other hand, F_2_BMet is effectively administered in micellar form based on the low content of the solubilizer CrEL (CrEL/EtOH/0.9% NaClaq 0.2:1:98.8 *v*/*v*/*v*), indicating appropriate tissue distribution in many types of tumors [12,15,22,24]. 15 min after administration it shows high blood concentration, which decreases with time, and good affinity to cancer tissues. It is characterized by a slightly longer stay in the body (about 65 h) [15,24]

**Figure 8:**
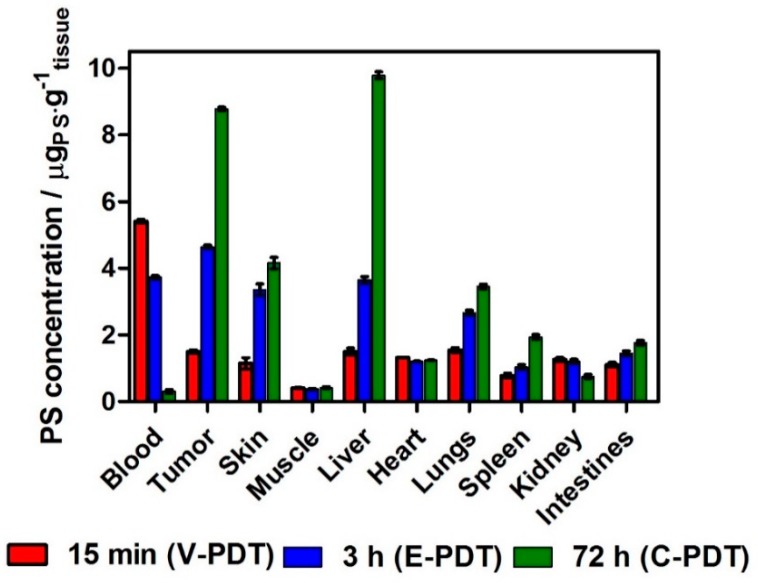
Biodistribution of Cl_2_BHep encapsulated in P123 micelles in organs and tissues extracted from BALB/c mice bearing CT26 tumors determined at various periods of time after the injection. The data are presented as mean ± SEM (*N* = 6).

The tissue distribution of Cl_2_BHep is described for the first time and is presented in Figure 8. The tissue distribution of Cl_2_BHep was determined using the extraction of tissue and biological fluids followed by homogenization and fluorescence measurements. 

The results clearly indicate that this hydrophobic PS can be favorable in C-PDT due to the significantly increased tumor accumulation in the prolonged incubation time (especially 72 h after PS injection). At this point, the PS concentration in tumors is four times higher than that obtained for 15 min, and the double of that for 3 h. The calculated tumor to muscle (T:M) ratios at 15 min., 3 h and 72 h are 3.6, 12.5 and 20.9, respectively. The tumor to skin (T:S) ratios are equal to 1.3, 1.38 and 2.11 at the same time. The highest T:M and T:S ratios at 72 h suggest a superior selectivity for Cl_2_BHep and a remarkable potential for cellular-targeted protocols with DLI = 72 h (C-PDT).

The summary of the photosensitizers’ properties crucial for optimization and appropriate choice of PDT protocols, as well as their systemic administration, are listed in Table 4.

### 3.10. PDT Efficacy

**Figure 9:**
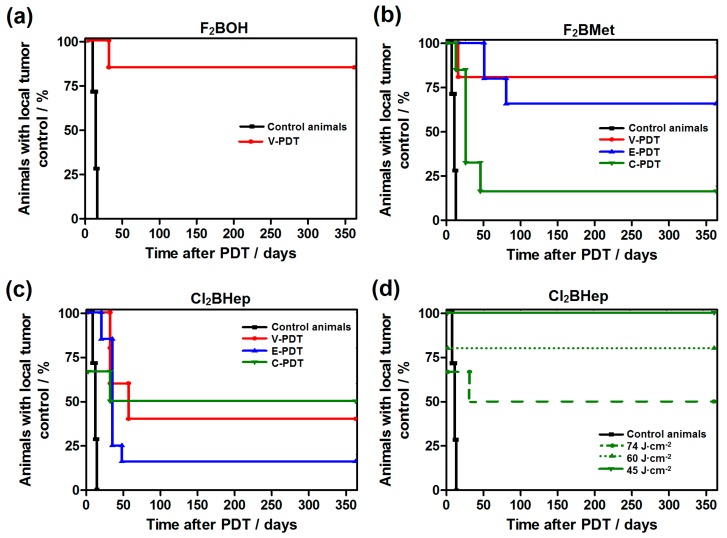
Kaplan–Meier curves of CT26 tumor regrowth after PDT treatment with: (**a**) F_2_BOH V-PDT; (**b**) F_2_BMet V-PDT, E-PDT and C-PDT; (**c**) Cl_2_BHep V-PDT, E-PDT and C-PDT; in all cases the tumor-bearing mice (*N* = 6–7) were irradiated with 74 J/cm^2^ dose of NIR laser light (130 mW, 748 nm) at 15 min, 3h or 72 h post-IV injection of 1.5 mg/kg drug dose. (**d**) presents the surviving data obtained for Cl_2_BHep-C-PDT after application of different laser light doses (light-dose de-escalation study) and 1.5 mg/kg drug dose.

We investigated the three basic PDT protocols, involving drug-to-light intervals of 15 min (V-PDT), 3 h (E-PDT) and 72 h (C-PDT) between the injection of the photosensitizer and the illumination. V-PDT aims at the destruction of tumor vessels, limiting the tumor nutrition and controlling tumor growth. In V-PDT, light is applied very soon after PS administration, while it is still within the vascular compartment. This protocol was tested for all the photosensitizers because all of them are mostly in the vascular compartment 15 min after i.v. administration. Alternatively, in tumor endothelial cells-targeted PDT (E-PDT), the drug-to-light interval is increased up to 3 h to take advantage of the accumulation of the photosensitizer both in the tumor microenvironment and tumor tissue. C-PDT with DLI = 72 h aims at the selective destruction of the tumor tissue while sparing the normal tissues. The latter two protocols were not tested for F2BOH because of its faster elimination from the organism. Figure 9 shows the Kaplan–Meier survival plots for all protocols with an indication of the percentage of animals with local tumor control.

Figure 9 shows that tumor growth control was observed in all groups. For F2BOH-V-PDT (Figure 9a), complete long-term remission was obtained for 85% of the treated animals. In the case of F_2_BMet (Figure 9b), the V-PDT protocol was also the most effective, resulting in about 80% of total cures. The F_2_BMet-E-PDT was also highly effective, resulting in 65% long-term cures. F_2_BMet C-PDT was less successful, with only 20% of cured mice. Interestingly, results obtained for the most hydrophobic photosensitizer Cl_2_BHep encapsulated in Pluronic P123 micelles indicate the highest percentage of cures, especially after C-PDT (Figure 9c,d). Cl_2_BHep C-PDT with 74 J/cm^2^ led to a very strong systemic inflammatory response that led to the death of some treated mice (35%) in the first 24 h after treatment (thus, survival analysis in Figure 8c starts at 65%). Subsequently, the light dose was de-escalated to obtain a safe protocol. C-PDT with a light dose of 60 J/cm2 remained lethal for 40% of the mice. A light dose of 45 J/cm2 no longer caused strong inflammatory reactions, and long-term cures were obtained for all of the animals. This is one of the most remarkable results with C-PDT protocols at such low light doses. The efficiency of Cl2BHep-C-PDT may be the result of the synergistic interaction of the following factors: (1) the formulation based on the PluronicP123 polymer; (2) appropriate accumulation of the photosensitizer in tumor tissue, which was demonstrated in a tissue distribution study; (3) efficient generation of ROSs, including type I mechanism. 

### 3.11. The Inflammatory Changes Observed after PDT

The PDT efficacy presented in the Kaplan–Meier plots indicates that the hydrophilic PS is most effective in V-PDT, the amphiphilic compound is suitable for both V- and E-PDT and the hydrophobic one shows superior activity in C-PDT. It is interesting to further explore the biological responses of these protocols and their possible connection with the very diverse therapeutic outcomes. 

**Figure 10:**
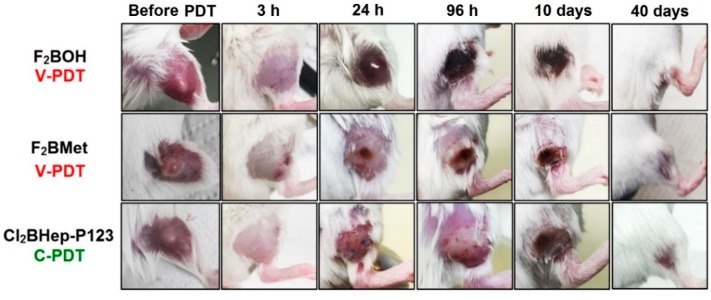
The differences in the inflammatory reaction occurring in BALB/c mice bearing CT26 tumors before and after PDT using optimal protocols with F2BOH, F2BMet, and Cl2BHep as the photosensitizer.

We observed differences in acute biological responses to PDT, such as edema and erythema, in vascular and cellular protocols (Figure 10). Edema usually appeared immediately after V-PDT and E-PDT, attained its maximum 24 h post-treatment and lasted until day 3–4. However, strong erythema and hyperemia were only present in animals subjected to F2BOH-V-PDT. For F2BMet-V-PDT the illumination area is characterized by large swelling, whereas after Cl2BHep-C-PDT, any strong edema is not observed. In the case of the F2BMet-V-PDT edema of the leg with the tumor, which was maximal at 3 h then decreased up to day 4, and then the progressive darkening of the tumor and scab formation are observed. For C-PDT, the inflammatory changes appear milder without significant edema, and tumor involution occurs over a prolonged period with the increase in necrotic area, as opposed to the acute effects observed with V-PDT. The different therapeutic protocols seem to generate a different immune response, which is likely to influence the therapeutic outcome. 

### 3.12. Examination of Inflammatory Cytokines Changes Induced by PDT

We further explored the inflammatory responses observed with different PSs and protocols evaluating changes in selected cytokines and proinflammatory mediators present in the blood before and 24 h after PDT using the Luminex xMAP technology, as shown in Scheme 2. The results in terms of observed trends in all tested biomarkers changes are presented in Table 5. These biomarkers can inform on the degree and mode of inflammation, infiltration of neutrophils, tumor cell death, as well as the destruction of the vasculature [61]. A first overview identifies common trends such as significant increases in IL-6 or KC, and tendencies to increase IL-10, MIP-2, MIP-1β or TNFα. However, there are also differences between the protocols.

**Figure 11:**
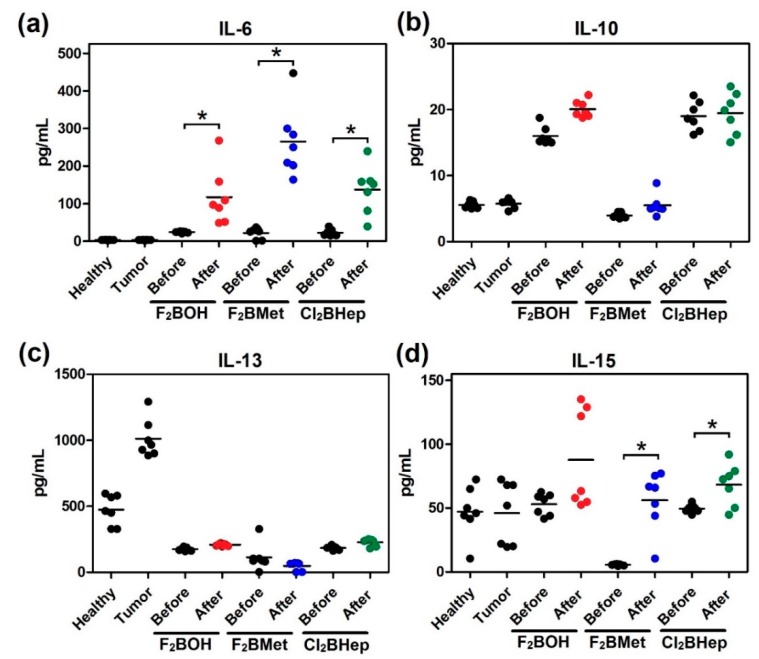
Concentrations of selected interleukins: (**a**) IL-6; (**b**) IL-10; (**c**) IL-13; (**d**) IL-15 in the serum of mice before and after optimal PDT protocol with F_2_BOH (V-PDT), F_2_BMet (V-PDT) and Cl_2_BHep (C-PDT) as well as healthy mice and mice bearing CT26 tumor controls. Luminex, N = 6–7; each dot represents one individual, line represents a mean; * *p* < 0.05.

Figure 11 focuses on the changes observed in the blood levels of interleukins. A significant increase in the concentration of IL-6 was observed in all cases (Figure 11a). The multidirectional nature of IL-6 interactions includes the participation in the activation of antigen-recognizing T lymphocytes (also LIF) as well as the production of acute-phase proteins indicating the early reaction in response to tissue damage. IL-6 expression was strongly and consistently enhanced in PDT-treated mice, although the role of IL-6 in PDT-induced inflammation may vary depending on the PS, protocol or type of tumor treated [61,62,63]. Increased IL-6 concentration in plasma can be considered as early and sensitive, although nonspecific, indicators of various inflammations, which in this case should be identified with the destruction of tumor tissue [64].

In the experimental groups that underwent PDT, these also exhibited a significant increase in IL-15 level, Figure 11d. In cancer immunotherapy, this cytokine is considered as a strong adjuvant, stimulating cytotoxicity and the proliferation of lymphocytes (cytotoxic, NKT) as well as the proliferation and secretion of antibodies activated by B cells [65]. IL15 is also an important growth factor for memory T lymphocytes, especially CD8+ [66]. The lack of unambiguous changes in IL-13 and especially in IL-10 (with the possible exception of (F_2_BOH), the V-PDT concentration after PDT (Figure 10b) using most protocols can be considered a good prognostic factor because it can ultimately lead to inhibition of the cell-type immune response and inflammatory processes [67]. In this respect, F_2_BOH V-PDT may not provide the best control of the inflammatory process. An increase in the concentration of CC-type chemokines (MCP-1, MIP1α, MIP1β) in the plasma after PDT can be seen (except for the F_2_BMet V-PDT and C-PDT protocol). A significant increase in the concentration of the inflammatory protein MCP-1 is evidence of increased inflammatory processes within the tumor microenvironment and the dominance of the necrotic pathway of cancer cell death and the infiltration of macrophages and monocytes into the tumor surrounding tissue [68,69].

**Figure 12:**
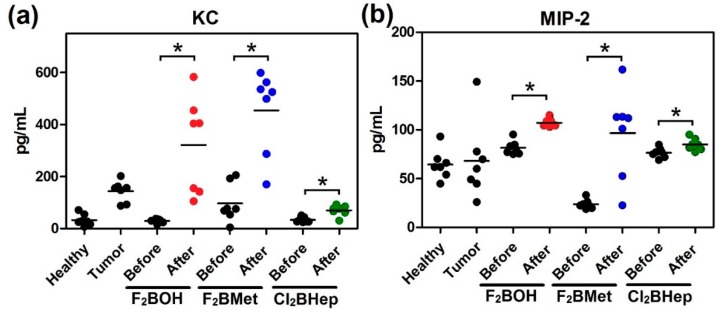
Concentrations of selected neutrophil chemokine attractants: (**a**) KC; (**b**) MIP-2; in the serum of mice before and after optimal PDT protocol with F_2_BOH (V-PDT), F_2_BMet (V-PDT) and Cl_2_BHep (C-PDT), as well as healthy mice and mice bearing CT26 tumor controls. Luminex, *N* = 6–7; each dot represents one individual, line represents a mean; * *p* < 0.05.

Several preclinical studies have suggested that suppression of long-term tumor growth following Photofrin-PDT is dependent upon the presence of neutrophils [70]. Neutrophil migration into the treated tumor is associated with an increase in the expression of the chemokines macrophage inflammatory protein MIP-2 and KC. Figure 12 shows that MIP-2 and KC increase significantly after PDT. In particular, V-PDT with both F_2_BOH and F_2_BMet causes a large increase in KC that is constitutively expressed and is believed to be involved in basal neutrophil migration. In contrast, MIP-2, the major chemokine released after F_2_BMet-V-PDT, is inducible and thought to mediate stress- or injury-induced neutrophil migration. 

The levels of KC and MIP-2 were unchanged in non-treated CT26 tumor-bearing mice and remained the comparable levels before PDT, which additionally indicates that the induction of MIP-2 and KC was not the result of a systemic mediator. Thus, bacteriochlorin-mediated V-PDT induced a time-dependent increase in two neutrophil attractant chemokines, MIP-2 and KC.

The optimal protocols with sulfonamide bacteriochlorin (F_2_BMet-V-PDT and Cl_2_BHep-C-PDT) are characterized by increased secretion of MIP-1, which is also involved in neutrophilic inflammation. Only in the case of F_2_BOH-V-PDT can the higher level of MIG (CXCL9) and IP-10 (CXCL10) be detected [71]. Importantly, these chemokines are known to exhibit antitumor activity through strong T-cell chemotaxis and anti-angiogenic (angiostatic) properties. Thus, due to the fact that their production is induced by interferons (IFNγ, inducible protein 10, IP-10, IFNγ-induced chemokine monokine 9, MIG), their increased plasma concentration after PDT indirectly indicates the activity of IFNγ [72]. 

**Figure 13:**
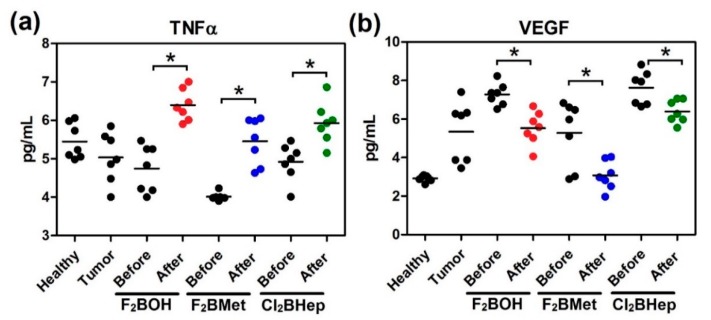
Concentrations of (**a**) TNFα; (**b**) VEGF in the serum of mice before and after optimal PDT protocol with F_2_BOH (V-PDT), F_2_BMet (V-PDT) and Cl_2_BHep (C-PDT) as well as healthy mice and mice bearing CT26 tumor controls. Luminex, *N* = 6–7; each dot represents one individual, line represents a mean; * *p* < 0.05.

Figure 13 shows how TNFα had increased in the blood of all groups that were subjected to the optimal scheme of PDT. It is widely known that TNFα upregulation may induce changes in the tumor vasculature and stimulate the antitumor immune response [73]. Moreover, these data can also suggest the activity of macrophages triggering in these cells Toll-like receptor (TLR)-based signal transduction activity resulting in the production of TNFα [61,62].

Last, but not least, we examined the vascular endothelial growth factor (VEGF). Figure 12b reveals a more significant decrease of VEGF after vascular-targeted treatment than after C-PDT. This observation suggests that the formation of new vessels within the tumor is inhibited, thereby stopping its further local progression and preventing distant metastases [74].

Taken together, the inflammatory changes observed after PDT and the patterns of inflammatory cytokines changes reveal that water-soluble photosensitizers such as F_2_BOH, generally lead to an increase in the serum level of these cytokines in V-PDT, when compared with less polar photosensitizers. This is consistent with the observation of strong erythema and hyperemia in animals subject to F_2_BOH-V-PDT. This may be related to the preferential association of hydrophilic photosensitizers with albumin, as opposed to the association of the less polar photosensitizers with lipoproteins. The tumor localization of albumin-bound and lipoprotein-bound photosensitizers is likely the cause of the difference in inflammatory reactions, because in the conditions of this study (same drug and light doses, comparable tumor concentration in V-PDT), all the photosensitizers produce similar oxidative stresses (similar light absorption and similar singlet oxygen quantum yields). Interestingly, the cytokine patterns revealed that V-PDT, E-PDT and C-PDT with F_2_BMet are surprisingly similar, although the photosensitizer concentration in the target tissue decreases appreciably with time. This suggests that the re-localization of the photosensitizer in the tumor may compensate for its lower concentration to yields-comparable inflammatory responses.

## 4. Summary and Conclusions

We determined physicochemical, spectroscopic and photochemical properties of three bacteriochlorins with widely different polarities (F2BOH, F2BMet, Cl2BHep), and related them with phototoxicity in vitro and PDT efficacy in prototypical PDT regimes. The most important difference between the studied compounds is their hydrophilicity/lipophilicity (logP) caused by a change from sulfonic group, to methylsulfonamide and to heptylsulfonamide ones, respectively. For the reasons discussed above, the chlorinated substituent has a higher singlet oxygen quantum yield (Φ_Δ_ = 0.63) than the fluorinated compounds (Φ_Δ_ ranged from 0.43 to 0.44), but this does not make Cl2BHep more phototoxic in vitro. The advantage of having chlorine atoms in the least soluble compound is to help avoid aggregation. The bulky chlorine atoms prevent the bacteriochlorins from sitting on the top of each other and facilitating dissolution.

PDT in vitro is influenced by differences in cellular uptake and subcellular localization. The pharmacokinetics and biodistribution of the photosensitizers determine the PDT regime (V-PDT, E-PDT, C-PDT) most adequate for each one of them. The most effective photosensitizers in V-PDT (DLI = 15 min) have hydrophilic or amphiphilic character, i.e. sulfonic and selected sulfonamide derivatives of bacteriochlorins (F2BOH, logP = −1.4; F2BMet logP = 1.9). The PDT protocol optimized for these photosensitizers led to >80% cure rates. More lipophilic photosensitizers (Cl2BHep, logP ≈ 4.5) proved to be more effective in protocols characterized by a longer DLI of 72 h. Such compounds, due to their limited solubility in the aqueous medium, need specialized formulations, such as encapsulation in Pluronic poloxamers micelles. This may be an additional challenge, but it comes with the advantages of increased stability, selectivity and overall efficacy. 

The different PDT regimes lead to different biological responses that can have distinct immunological components and contribute to the overall PDT efficacy. Various degree of local inflammation is observed after different PDT regimes. Multiplex analysis of selected cytokines present in the blood before and 24 h after the PDT showed increased IL-6 concentrations in plasma after PDT, as expected from the inflammations observed. The enhanced concentration of KC and MIP-2 chemokines after PDT suggest that tumor response may involve an influx of neutrophils to the tumor. The optimal protocols with sulfonamide bacteriochlorin (F2BMet-V-PDT and Cl2BHep-C-PDT) are characterized by increased secretion of MIP-1, which is also involved in neutrophilic inflammation. 

The selected optimal protocols indicate the increased level of TNFα activity and decreased VEGF, especially after anti-vascular treatment. Further studies are necessary to fully understand how the changes in these biomarkers after PDT contribute to mounting an adaptive immune response capable of inducing immunological memory and recognition of tumor cells surviving the local PDT treatment.

## Figures and Tables

**Table 1:** Inflammatory cytokines/chemokines analyzed in this work.

Cytokine/Chemokine	Abbreviation
Interleukin 6	IL-6
Interleukin 10	IL-10
Interleukin 13	IL-13
Interleukin 15	IL-15
Keratinocyte-derived chemokine/C-X-C motif chemokine receptor 1 (CXCL1)	KC
Macrophage inflammatory protein 2 (MIP-2)/C-X-C motif chemokine ligand 2 (CXCL2)	MIP-2
The murine homolog of ENA-78/C-X-C motif chemokine ligand 5 (CXCL5)	LIX
Leukemia inhibitory factor (interleukin 6 class cytokine)	LIF
Monocyte chemoattractant protein-1	MCP-1
Macrophage inflammatory protein 1 alpha	MIP-1α
Macrophage inflammatory protein 1 beta	MIP-1β
Interferon gamma-induced protein 10/C-X-C motif chemokine 10 (CXCL10)	IP-10
Monokine induced by interferon-gamma/C-X-C motif chemokine ligand 9 (CXCL9)	MIG
Granulocyte-macrophage colony-stimulating factor	GM-CSF
Tumor necrosis factor-alpha	TNFα
Vascular endothelial growth factor	VEGF

**Table 2:** Photophysical properties of investigated bacteriochlorins determined in ethanol (EtOH) and their lipophilicity (logP).

PS	λ_max_/nm; ε_NIR_/10^3^M^−1^cm^−1^	λ_fl_/nm	Φ_F_	τ_F_/ns	Φ_pb_/10^−6^	Φ_Δ_	τ_T_/ns	logP
F_2_BOH	743; 70.0 [24]	764	0.023	3.90	1620 ^a^	0.44 [49]	268 [23]	−1.4
F_2_BMet	743; 100 [23]	768	0.138	3.91	75 ^a^	0.43 [49]	216 [23]	1.9
Cl_2_BHep	746; 76.0 [49]	752	0.008	1.05	55 ^a^	0.63 [23]	295 [49]	4.5

^a^ determined in PBS (0.5% DMSO).

**Table 3:** Lethal light doses (LLD_50_) of NIR light required to kill 50% of CT26 determined from surviving curves obtained in phototoxicity studies.

	Photosensitizer				
	F_2_BOH	F_2_BOH V-PDE	F_2_BMet	Cl_2_BHep	Cl_2_BHep-P123
**LLD_50_/J·cm^−2^**	−	4.81	4.94	>20	5.66

**Table 4:** Properties of investigated bacteriochlorin-based photosensitizers and those used in clinical practice, crucial for determining the photodynamic therapy (PDT) protocols in vivo.

Photosensitizer	logP	Tumor uptake	PK/Circulation Time	DLI	Formulation
F_2_BOH [24]	−1.4	3.69 pM per cell	44 h	15 min (V-PDT)	0.9% NaCl_aq_/PBS
F_2_BMet [15,22]	1.9	24 pM per cell	65 h	15 min (V-PDT)	CrEL:PBS
Cl_2_BHep	4.5	7.5 pM per cell	< 96 h	72 h (C-PDT)	Pluronic P123
Tookad Soluble [58]	>1.0	max. 50 pg/mg protein	max 1h	>15 min (V-PDT)	0.9% NaCl_aq_
Photofrin [59,60]	<0.15	max. 1.6 µg/mg protein	144 h	6–24 h (C-PDT)	Saline + 5% glucose

**Table 5:** The trend in changes of selected cytokines and chemokines determined after bacteriochlorin-based PDT. The analysis was performed using Luminex multiplexing assay in the serum of individual mice before and after PDT (N = 6–7).

PS	F2BOH	F2BMet			Cl2BHep		
Cytokine	V-PDT	V-PDT	E-PDT	C-PDT	V-PDT	E-PDT	C-PDT
IL-6	⬆⬆	⬆	⬆	⬆	⬆	⬆⬆	⬆
IL-10	⇧	−	⇧	⇧	⇧	−	⬆
IL-13	⬆	⇩	⇩	−	⬆	⬆	⇧
IL-15	⇧	⇧	−	⇧	⬆	⬆	⬆
KC	⬆	⬆	⬆	⬆	⬆	⬆⬆	⬆
MIP-2	⬆	−	⬆	⬆	−	⬆	⇧
LIX	⬆	⇩	⇩	−	−	⇩	⇩
LIF	⇧	⇧	⇧	−	−	−	⬇
MCP-1	⬆	−	⬆	⬆	⬆	⇧	⬆
MIP-1α	−	⬆	⬆	−	−	⇧	⇩
MIP-1β	⇩	−	⬆	−	−	⇧	⇩
IP-10	⬆	−	⬆	−	⬇	−	⬆
MIG	⬆	⬆	⬇	−	⬆	−	−
GM-CSF	⇧	−	⇩	−	−	−	−
VEGF	⬇	⬇	−	−	⬇	−	⇩
TNFα	⬆	⬆	⇧	−	−	−	⬆

⬆, ⬇ indicated significantly (min two-times) increased or decreased the level of each biomarker, ⇧ and ⇩ are symbols for the non-significant (higher or lower) tendency in the concentration of biomarker. The tendencies were estimated in comparison to values obtained in the serum of the same individual animals before PDT.

## References

[1] Kessel D. (2019). Photodynamic Therapy: A Brief History. J. Clin. Med..

[2] Dabrowski J.M., Arnaut L.G. (2015). Photodynamic therapy (PDT) of cancer: From local to systemic treatment. Photochem. Photobiol. Sci..

[3] Dąbrowski J.M. (2017). Reactive oxygen species in photodynamic therapy: Mechanisms of their generation and potentiation. Advances in Inorganic Chemistry.

[4] Agostinis P., Berg K., Cengel K.A., Foster T.H., Girotti A.W., Gollnick S.O., Hahn S.M., Hamblin M.R., Juzeniene A., Kessel D. (2011). Photodynamic therapy of cancer: An update. CA Cancer J. Clin..

[5] Krzykawska-Serda M., Dąbrowski J.M., Arnaut L.G., Szczygieł M., Urbańska K., Stochel G., Elas M. (2014). The role of strong hypoxia in tumors after treatment in the outcome of bacteriochlorin-based photodynamic therapy. Free Radic. Biol. Med..

[6] Karwicka M., Pucelik B., Gonet M., Elas M., Dąbrowski J.M. (2019). Effects of Photodynamic Therapy with Redaporfin on Tumor Oxygenation and Blood Flow in a Lung Cancer Mouse Model. Sci. Rep..

[7] Donohoe C., Senge M.O., Arnaut L.G., Gomes-da-Silva L.C. (2019). Cell death in photodynamic therapy: From oxidative stress to anti-tumor immunity. Biochim. Biophys. Acta Rev. Cancer.

[8] Chen B., Pogue B.W., Hoopes P.J., Hasan T. (2006). Vascular and cellular targeting for photodynamic therapy. Crit. Rev. Eukaryot. Gene Expr..

[9] García-Díaz M., Kawakubo M., Mroz P., Sagristà M.L., Mora M., Nonell S., Hamblin M.R. (2012). Cellular and vascular effects of the photodynamic agent temocene are modulated by the delivery vehicle. J. Control. Release.

[10] Kiesslich T., Krammer B., Plaetzer K. (2006). Cellular mechanisms and prospective applications of hypericin in photodynamic therapy. Curr. Med. Chem..

[11] Krammer B. (2001). Vascular effects of photodynamic therapy. Anticancer Res..

[12] Pucelik B., Arnaut L.G., Stochel G.Y., Dabrowski J.M. (2016). Design of Pluronic-based formulation for enhanced redaporfin-photodynamic therapy against pigmented melanoma. ACS Appl. Mater. Interfaces.

[13] Dąbrowski J.M., Pucelik B., Regiel-Futyra A., Brindell M., Mazuryk O., Kyzioł A., Stochel G., Macyk W., Arnaut L.G. (2016). Engineering of relevant photodynamic processes through structural modifications of metallotetrapyrrolic photosensitizers. Coord. Chem. Rev..

[14] Castano A.P., Demidova T.N., Hamblin M.R. (2004). Mechanisms in photodynamic therapy: Part one—photosensitizers, photochemistry and cellular localization. Photodiagnosis Photodyn. Ther..

[15] Saavedra R., Rocha L.B., Dąbrowski J.M., Arnaut L.G. (2014). Modulation of biodistribution, pharmacokinetics, and photosensitivity with the delivery vehicle of a bacteriochlorin photosensitizer for photodynamic therapy. ChemMedChem.

[16] Jori G., Reddi E. (1993). The role of lipoproteins in the delivery of tumour-targeting photosensitizers. Int. J. Biochem..

[17] Huang Y.-Y., Mroz P., Zhiyentayev T., Sharma S.K., Balasubramanian T., Ruzié C., Krayer M., Fan D., Borbas K.E., Yang E. (2010). In vitro photodynamic therapy and quantitative structure—Activity relationship studies with stable synthetic near-infrared-absorbing bacteriochlorin photosensitizers. J. Med. Chem..

[18] Oliveira C.S., Turchiello R., Kowaltowski A.J., Indig G.L., Baptista M.S. (2011). Major determinants of photoinduced cell death: Subcellular localization versus photosensitization efficiency. Free Radic. Biol. Med..

[19] Brandis A., Mazor O., Neumark E., Rosenbach-Belkin V., Salomon Y., Scherz A. (2005). Novel Water-soluble Bacteriochlorophyll Derivatives for Vascular-targeted Photodynamic Therapy: Synthesis, Solubility, Phototoxicity and the Effect of Serum Proteins. Photochem. Photobiol..

[20] Azzouzi A.R., Barret E., Moore C.M., Villers A., Allen C., Scherz A., Muir G., de Wildt M., Barber N.J., Lebdai S. (2013). TOOKAD® S oluble vascular-targeted photodynamic (VTP) therapy: Determination of optimal treatment conditions and assessment of effects in patients with localised prostate cancer. BJU Int..

[21] Rocha L.B., Gomes-da-Silva L.C., Dąbrowski J.M., Arnaut L.G. (2015). Elimination of primary tumours and control of metastasis with rationally designed bacteriochlorin photodynamic therapy regimens. Eur. J. Cancer.

[22] Rocha L., Schaberle F., Dąbrowski J., Simões S., Arnaut L. (2015). Intravenous single-dose toxicity of redaporfin-based photodynamic therapy in rodents. Int. J. Mol. Sci..

[23] Arnaut L.G., Pereira M.M., Dąbrowski J.M., Silva E.F., Schaberle F.A., Abreu A.R., Rocha L.B., Barsan M.M., Urbańska K., Stochel G. (2014). Photodynamic therapy efficacy enhanced by dynamics: The role of charge transfer and photostability in the selection of photosensitizers. Chem. A Eur. J..

[24] Luz A.F., Pucelik B., Pereira M.M., Dąbrowski J.M., Arnaut L.G. (2018). Translating phototherapeutic indices from in vitro to in vivo photodynamic therapy with bacteriochlorins. Lasers Surg. Med..

[25] Dąbrowski J.M., Arnaut L.G., Pereira M.M., Urbańska K., Simões S., Stochel G., Cortes L. (2012). Combined effects of singlet oxygen and hydroxyl radical in photodynamic therapy with photostable bacteriochlorins: Evidence from intracellular fluorescence and increased photodynamic efficacy in vitro. Free Radic. Biol. Med..

[26] Dąbrowski J.M., Urbanska K., Arnaut L.G., Pereira M.M., Abreu A.R., Simões S., Stochel G. (2011). Biodistribution and photodynamic efficacy of a water-soluble, stable, halogenated bacteriochlorin against melanoma. ChemMedChem.

[27] Pereira M.M., Monteiro C.J., Simões A.V., Pinto S.M., Arnaut L.G., Sá G.F., Silva E.F., Rocha L.B., Simões S., Formosinho S.J. (2009). Synthesis and photophysical properties of amphiphilic halogenated bacteriochlorins: New opportunities for photodynamic therapy of cancer. J. Porphyr. Phthalocyanines.

[28] Dąbrowski J.M., Arnaut L.G., Pereira M.M., Urbańska K., Stochel G. (2012). Improved biodistribution, pharmacokinetics and photodynamic efficacy using a new photostable sulfonamide bacteriochlorin. MedChemComm.

[29] Pandey R.K., Potter W.R., Dougherty T.J. (2007). Fluorinated Photosensitizers Related to Chlorins and Bacteriochlorins for Photodynamic Therapy. U.S. Patent.

[30] Pandey S.K., Gryshuk A.L., Graham A., Ohkubo K., Fukuzumi S., Dobhal M.P., Zheng G., Ou Z., Zhan R., Kadish K.M. (2003). Fluorinated photosensitizers: Synthesis, photophysical, electrochemical, intracellular localization, in vitro photosensitizing efficacy and determination of tumor-uptake by 19F in vivo NMR spectroscopy. Tetrahedron.

[31] Goslinski T., Piskorz J. (2011). Fluorinated porphyrinoids and their biomedical applications. J. Photochem. Photobiol. C Photochem. Rev..

[32] Gryshuk A.L., Graham A., Pandey S.K., Potter W.R., Missert J.R., Oseroff A., Dougherty T.J., Pandey R.K. (2002). A First Comparative Study of Purpurinimide-based Fluorinated vs. Nonfluorinated Photosensitizers for Photodynamic Therapy. Photochem. Photobiol..

[33] Naumann K. (1999). Influence of chlorine substituents on biological activity of chemicals. J. Prakt. Chem..

[34] Naumann K. (2000). Influence of chlorine substituents on biological activity of chemicals: A review. Pest Manag. Sci. Former. Pestic. Sci..

[35] Castano A.P., Mroz P., Hamblin M.R. (2006). Photodynamic therapy and anti-tumour immunity. Nat. Rev. Cancer.

[36] Korbelik M., Dougherty G.J. (1999). Photodynamic therapy-mediated immune response against subcutaneous mouse tumors. Cancer Res..

[37] Gollnick S.O., Brackett C.M. (2010). Enhancement of anti-tumor immunity by photodynamic therapy. Immunol. Res..

[38] Wyld L., Reed M., Brown N. (2001). Differential cell death response to photodynamic therapy is dependent on dose and cell type. Br. J. Cancer.

[39] Nowis D., Stokłosa T., Legat M., Issat T., Jakóbisiak M., Gołąb J. (2005). The influence of photodynamic therapy on the immune response. Photodiagnosis Photodyn. Ther..

[40] Garg A.D., Nowis D., Golab J., Agostinis P. (2010). Photodynamic therapy: Illuminating the road from cell death towards anti-tumour immunity. Apoptosis.

[41] Gomes-da-Silva L.C., Zhao L., Bezu L., Zhou H., Sauvat A., Liu P., Durand S., Leduc M., Souquere S., Loos F. (2018). Photodynamic therapy with redaporfin targets the endoplasmic reticulum and Golgi apparatus. EMBO J..

[42] Brackett C.M., Gollnick S.O. (2011). Photodynamic therapy enhancement of anti-tumor immunity. Photochem. Photobiol. Sci..

[43] Korbelik M., Naraparaju V., Yamamoto N. (1997). Macrophage-directed immunotherapy as adjuvant to photodynamic therapy of cancer. Br. J. Cancer.

[44] Sun J., Cecic I., Parkins C.S., Korbelik M. (2002). Neutrophils as inflammatory and immune effectors in photodynamic therapy-treated mouse SCCVII tumours. Photochem. Photobiol. Sci..

[45] Garg A.D., Dudek A.M., Agostinis P. (2013). Cancer immunogenicity, danger signals, and DAMPs: What, when, and how?. Biofactors.

[46] Garg A.D., Krysko D.V., Vandenabeele P., Agostinis P. (2011). DAMPs and PDT-mediated photo-oxidative stress: Exploring the unknown. Photochem. Photobiol. Sci..

[47] Krysko D.V., Garg A.D., Kaczmarek A., Krysko O., Agostinis P., Vandenabeele P. (2012). Immunogenic cell death and DAMPs in cancer therapy. Nat. Rev. Cancer.

[48] Pereira M.M., Monteiro C.J., Simões A.V., Pinto S.M., Abreu A.R., Sá G.F., Silva E.F., Rocha L.B., Dąbrowski J.M., Formosinho S.J. (2010). Synthesis and photophysical characterization of a library of photostable halogenated bacteriochlorins: An access to near infrared chemistry. Tetrahedron.

[49] Silva E.F.F.D. (2013). Transparent Photochemistry: Infrared Photosensitizers and Singlet Oxygen Microscopy.

[50] Huang Y.Y., Balasubramanian T., Yang E., Luo D., Diers J.R., Bocian D.F., Lindsey J.S., Holten D., Hamblin M.R. (2012). Stable synthetic bacteriochlorins for photodynamic therapy: Role of dicyano peripheral groups, central metal substitution (2H, Zn, Pd), and Cremophor EL delivery. ChemMedChem.

[51] Vakrat-Haglili Y., Weiner L., Brumfeld V., Brandis A., Salomon Y., Mcllroy B., Wilson B.C., Pawlak A., Rozanowska M., Sarna T. (2005). The microenvironment effect on the generation of reactive oxygen species by Pd− bacteriopheophorbide. J. Am. Chem. Soc..

[52] Ashur I., Goldschmidt R., Pinkas I., Salomon Y., Szewczyk G., Sarna T., Scherz A. (2009). Photocatalytic Generation of Oxygen Radicals by the Water-Soluble Bacteriochlorophyll Derivative WST11, Noncovalently Bound to Serum Albumin. J. Phys. Chem. A.

[53] Madar-Balakirski N., Tempel-Brami C., Kalchenko V., Brenner O., Varon D., Scherz A., Salomon Y. (2010). Permanent occlusion of feeding arteries and draining veins in solid mouse tumors by vascular targeted photodynamic therapy (VTP) with Tookad. PLoS ONE.

[54] Pucelik B., Paczyński R., Dubin G., Pereira M.M., Arnaut L.G., Dąbrowski J.M. (2017). Properties of halogenated and sulfonated porphyrins relevant for the selection of photosensitizers in anticancer and antimicrobial therapies. PLoS ONE.

[55] Moan J., Berg K., Kvam E., Western A., Malik Z., Rück A., Schneckenburger H. (2007). Intracellular localization of photosensitizers. Ciba Foundation Symposium 146-Photosensitizing Compounds: Their Chemistry, Biology and Clinical Use: Photosensitizing Compounds: Their Chemistry, Biology and Clinical Use: Ciba Foundation Symposium 146.

[56] Kessel D., Luo Y. (2001). Intracellular sites of photodamage as a factor in apoptotic cell death. J. Porphyr. Phthalocyanines.

[57] Sobczynski J., Kristensen S., Berg K. (2014). The influence of Pluronics nanovehicles on dark cytotoxicity, photocytotoxicity and localization of four model photosensitizers in cancer cells. Photochem. Photobiol. Sci..

[58] Mazor O., Brandis A., Plaks V., Neumark E., Rosenbach-Belkin V., Salomon Y., Scherz A. (2005). WST11, A Novel Water-soluble Bacteriochlorophyll Derivative; Cellular Uptake, Pharmacokinetics, Biodistribution and Vascular-targeted Photodynamic Activity Using Melanoma Tumors as a Model. Photochem. Photobiol..

[59] Hajri A., Wack S., Meyer C., Smith M., Leberquier C., Kedinger M., Aprahamian M. (2002). In Vitro and In Vivo Efficacy of Photofrin® and Pheophorbide a, a Bacteriochlorin, in Photodynamic Therapy of Colonic Cancer Cells. Photochem. Photobiol..

[60] Li L.-B., Luo R.-C. (2009). Effect of drug–light interval on the mode of action of Photofrin photodynamic therapy in a mouse tumor model. Lasers Med Sci..

[61] Gollnick S., Evans S., Baumann H., Owczarczak B., Maier P., Vaughan L., Wang W., Unger E., Henderson B. (2003). Role of cytokines in photodynamic therapy-induced local and systemic inflammation. Br. J. Cancer.

[62] Evans S., Matthews W., Perry R., Fraker D., Norton J., Pass H.I. (1990). Effect of photodynamic therapy on tumor necrosis factor production by murine macrophages. JNCI J. Natl. Cancer Inst..

[63] Gollnick S.O., Liu X., Owczarczak B., Musser D.A., Henderson B.W. (1997). Altered expression of interleukin 6 and interleukin 10 as a result of photodynamic therapy in vivo. Cancer Res..

[64] Liu W., Oseroff A.R., Baumann H. (2004). Photodynamic therapy causes cross-linking of signal transducer and activator of transcription proteins and attenuation of interleukin-6 cytokine responsiveness in epithelial cells. Cancer Res..

[65] Jakobisiak M., Golab J., Lasek W. (2011). Interleukin 15 as a promising candidate for tumor immunotherapy. Cytokine Growth Factor Rev..

[66] Teague R.M., Sather B.D., Sacks J.A., Huang M.Z., Dossett M.L., Morimoto J., Tan X., Sutton S.E., Cooke M.P., Öhlén C. (2006). Interleukin-15 rescues tolerant CD8+ T cells for use in adoptive immunotherapy of established tumors. Nat. Med..

[67] Sato T., Terai M., Tamura Y., Alexeev V., Mastrangelo M.J., Selvan S.R. (2011). Interleukin 10 in the tumor microenvironment: A target for anticancer immunotherapy. Immunol. Res..

[68] Hoshino Y., Hatake K., Kasahara T., Takahashi Y., Ikeda M., Tomizuka H., Ohtsuki T., Uwai M., Mukaida N., Matsushima K. (1995). Monocyte chemoattractant protein-1 stimulates tumor necrosis and recruitment of macrophages into tumors in tumor-bearing nude mice: Increased granulocyte and macrophage progenitors in murine bone marrow. Exp. Hematol..

[69] De Filippo K., Dudeck A., Hasenberg M., Nye E., van Rooijen N., Hartmann K., Gunzer M., Roers A., Hogg N. (2013). Mast cell and macrophage chemokines CXCL1/CXCL2 control the early stage of neutrophil recruitment during tissue inflammation. Blood.

[70] Kousis P.C., Henderson B.W., Maier P.G., Gollnick S.O. (2007). Photodynamic therapy enhancement of antitumor immunity is regulated by neutrophils. Cancer Res..

[71] Tokunaga R., Zhang W., Naseem M., Puccini A., Berger M.D., Soni S., McSkane M., Baba H., Lenz H.-J. (2018). CXCL9, CXCL10, CXCL11/CXCR3 axis for immune activation–a target for novel cancer therapy. Cancer Treat. Rev..

[72] Gorbachev A., Kish D., Fairchild R. (2013). Induction of CXCL9/Mig expression in the tumor microenvironment promotes protective antitumor immune responses. Cancer Res..

[73] Kawczyk-Krupka A., Czuba Z., Szliszka E., Król W., Sieroń A. (2011). The role of photosensitized macrophages in photodynamic therapy. Oncol. Rep..

[74] Solban N., Pål S.K., Alok S.K., Sung C.K., Hasan T. (2006). Mechanistic investigation and implications of photodynamic therapy induction of vascular endothelial growth factor in prostate cancer. Cancer Res..

